# Transcriptome analysis provides insights into the response of *Lotus corniculatus* roots to low-phosphorus stress

**DOI:** 10.3389/fpls.2023.1089380

**Published:** 2023-03-01

**Authors:** Xin Zhao, Ke-ke Chen, Lei-ting Wang, Li-Li Zhao, Pu-Chang Wang

**Affiliations:** ^1^ College of Animal Science, Guizhou University, Guiyang, China; ^2^ School of Life Sciences, Guizhou Normal University, Guiyang, China

**Keywords:** *Lotus corniculatus*, low-phosphorus stress, physiological, morphological, transcriptomic

## Abstract

**Introduction:**

A lack of soil phosphorus (P) is a principal factor restricting the normal growth of *Lotus corniculatus* in the karst area of Guizhou Province, China, but the response mechanism of *L. corniculatus* under low-phosphorus stress remains unclear.

**Methods:**

Therefore, we treated two selected *L. corniculatus* lines (low-P-intolerant line 08518 and low-P-tolerant line 01549) from 13 *L. corniculatus* lines with normal phosphorus (0.5 mmol/L KH_2_PO_4_, NP) and low phosphorus (0.005 mmol/L KH_2_PO_4_, LP) concentrations to study changes in morphological, physiological and transcriptome data under low-phosphorus stress.

**Results:**

The low-P-tolerant line 01549 exhibited better performance under low-phosphorus stress. Compared with the NP treatment, all root morphological indicators of the low-P-tolerant line 01549 increased, and those of the low-P-intolerant line 08518 decreased under low-P stress. Compared with the NP treatment, acid phosphatase (ACP), catalase (CAT), superoxide dismutase (SOD), and peroxidase (POD) activities, and the malondialdehyde (MDA), soluble sugar (SS), soluble protein (SP) and proline (Pro) contents of the two *L. corniculatus* lines increased under low-P stress. A transcriptome analysis of *L. corniculatus* showed that a total of 656 and 2243 differentially expressed genes (DEGs) were identified in line 01549 and line 08518, respectively. Meanwhile, the main pathways, such as carbohydrate metabolism, acid phosphatases, phosphate transporters and biosynthesis of secondary metabolites, as well as related genes were also screened by performing a KEGG enrichment analysis.

**Discussion:**

The findings provide an essential point of reference for studying the physiological and molecular mechanism of the response to low-P stress in *L. corniculatus*.

## Introduction

Phosphorus (P), one of the essential elements for plants, plays a key role not only in promoting plant growth and development, but also in regulating physiological metabolism and resistance to stress (Ding et al., 2021; Zipfel and Oldroyd, 2017). The soil P concentration is high, but about 80% of the phosphorus is organic phosphorus which is difficult to be used by plants, while inorganic phosphate in soil is easily fixed by iron, aluminum and calcium ions to form insoluble phosphate which cannot be used by plants directly (Chiou et al., 2006). Usually, the concentration of available phosphate in soil does not exceed 2.0 uM, and even in fertile soils it rarely exceeds 10 uM, far from meeting the needs of normal plant growth(Yuan and Liu, 2008). In order to improve the efficiency of P acquisition and utilization by plants, many scholars have studied the mechanisms of plant adaptation and tolerance to low-P stress(Nilsson et al., 2010; Ried et al., 2021).

Plants have been shown to have evolved a range of complex and tightly controlled mechanisms for maintaining cellular Pi concentrations (Tesfaye et al., 2007). One of the most important mechanisms is the ability of the plant root system to improve phosphorus uptake from the soil and autologous phosphorus utilization through a series of collaborative processes involving changes in plant morphology, physiology and molecular biology (Verma et al., 2021). Some studies have reported that plant biomass decreases significantly under low-P conditions and that the root surface area, total root number, lateral root length, number of lateral roots and root volume increase, expanding the area of P uptake by the root system (Hernández et al., 2007; Raya-González et al., 2021). O'Rourke et al. (2013) found that when P is deficient in the soil, white lupin forms cluster roots, while the row roots secrete organic acids to activate the fixed P in the soil, thereby increasing the effective P concentration in the medium. Simultaneously, the plant activates the fixed phosphorus in the soil through its own physiological and biochemical changes, such as the secretion of organic acids, and increases the effective phosphorus content through chemical action (Plaxton and Tran, 2011; Ciereszko et al., 2011). Li et al. (2016) found that *Leymus chinensis* protected PSII against Pi-induced photooxidative stress in the organism by enhancing the activity of anti-reactive oxygenase. Under P-deprived conditions, chlorophyll contents, soluble protein contents and root vigor are reduced, while malondialdehyde (MDA), soluble sugar and free proline contents are increased, as are peroxidase (POD), superoxide dismutase (SOD) and catalase (CAT) activities (Rohman et al., 2019).

The morphological, physiological, and biochemical changes exhibited by plants under low-P stress are achieved by the synergistic expression of phosphate starvation-response (PSR) genes in plant tissues (Zhang H. et al., 2014). Sun and Zheng (2022) found *ZmPHT1;1*, *ZmPHT1;9* and Zmphytase 2 genes play a key role in phosphorus recovery in maize. Hu et al. (2021) found P deficit increases the expression of genes related to glycerolipid synthesis, while the expression of genes related to phospholipid breakdown is inhibited. An increase in the expression of the genes encoding the PSII protein complex, the cytochrome b/f protein complex, the PSI protein complex, photosynthetic electron transport proteins, and several members of the F-type ATPase family was observed. Müller et al. (2007) found that chronic low-P stress affected primary and secondary plant metabolism, disrupted the balance of carbon metabolism, inhibited the binding of proteins and chlorophyll a or b, and reduced the photosynthetic capacity of plants, while the expression of genes related to sucrose and glucose metabolism were upregulated. Jeong et al. (2017) studied the rice transcriptome under low-P stress and found that kinase and phosphotransferase-related genes were upregulated, and the expression of genes involved in ribosome composition, transcription and translation was downregulated. In addition, in-depth transcriptomics analyses of oil palm (Kong et al., 2021), barley (Ren et al., 2018), wheat (Wang et al., 2019) and sorghum (Zhang J. et al., 2019) under low-P stress have also been performed, and the corresponding drought stress-responsive genes have been identified.

*Lotus corniculatus* is a perennial herb of the genus *Lotus* with strong tolerance of cold, barren and drought, developed root system, creeping stem and branch growth, strong grip, wide coverage, and is an excellent grass species for karst ecological environment management (Knežević et al., 2021). Second, the stems and leaves of *L. corniculatus* are soft and juicy, rich in carbohydrates, with high yield, good palatability, a high saponin content and enrichment of condensed tannins, which may reduce the occurrence of swelling disease in livestock (Antonelli et al., 2021). Its root system has many rhizomes and has good nitrogen fixation, which can increase soil organic matter and improve soil structure, and is a high-quality forage plant resource and green manure (Jiang et al., 2021). Genetic diversity analysis of accession resources is the basis for new variety selection and breeding, and since the 1980s, some cultivars of *L. corniculatus* have been introduced from abroad in China, and production trials have shown that these varieties have strong adaptability and high production performance and are widely planted in northwest China (Xie et al., 2023). In the karst region of southwest China, the research on the selection and breeding of varieties of *L. corniculatus* is not systematic, and there are few new varieties of *L. corniculatus*. It is of great practical significance for the large-scale cultivation of *L. corniculatus* and the development of local ecological animal husbandry in karst mountainous areas of southwest China by introducing new excellent *L. corniculatus* line resources and breeding accession varieties suitable for different ecological zones and uses. Moreover, the karst region of southwest China with severe regional rock desertification and effective phosphorus deficiency, and low phosphorus concentrations can impair plant growth and development (Baghbani-Arani et al., 2021; Zhao et al., 2022a), it is of great significance and application value to cultivate phosphorus-efficient plants and improve the utilization of phosphorus in soil by plants. Therefore, in the present study, we used the line resources of *L. corniculatus* as the object of the study and established two treatments, normal and low P concentrations, to screen two line species of *L. corniculatus* that are tolerant or sensitive to low P concentrations. The aim was to elucidate the adaptive mechanisms and identify the genes regulating the low-P tolerance in the two genotypes of *L. corniculatus*. And it is intended to provide a preliminary reference for the innovative utilization of excellent *L. corniculatus* line resources and the selection and breeding of new varieties.

## Materials and methods

### Accession selection

We conducted sand culture experiments to evaluate the P tolerance of 13 *L. corniculatus* lines (**Table S1**) provided by the National Germplasm Forage Medium Term Repository, Institute of Grassland Research, Chinese Academy of Agricultural Sciences. The experiment was performed at Guizhou University College of Animal Science from May to August 2021. For the tested *L. corniculatus* lines, 4-8 cm cuttings were planted in different pots, the plants were grown and divided, the roots were washed with distilled water, and the uniformly growing *L. corniculatus* lines was selected and planted in seedling trays filled with quartz sand (width: high: long = 28 cm: 7.5 cm: 54 cm) and precultured with 15 seedlings per tray in a nutrient solution on 14 h/10 h light/dark cycle at 26°C/22°C. Three trays were included for one treatment. The nutrient solution was prepared with deionised water, which was formulated as described in our previous research article (Zhao et al., 2022b). Plants were incubated with the complete nutrient solution for one week and then divided into two treatment groups consisting of normal (0.5 mmol/L KH_2_PO_4_, NP) and low phosphorus (0.005 mmol/L KH_2_PO_4_, LP) concentrations, with the nutrient solution replenished every 2 d. KH_2_PO_4_ was used as the phosphorus source, the K^+^ concentration was balanced by K_2_SO_4_, and the pH of the nutrient solution was adjusted to 6.00 using NaOH and H_2_SO_4_. After 21 d, root tissues from the control and low-P stress groups were collected to measure their growth characteristics and whole plant P concentration.

### Determination of morphological indices

A straightedge was used to measure the plant height. The aboveground and belowground sections of the plant were separated and the fresh weights were measured before these parts were placed in an oven at 105°C for 30 min and dried to a constant weight at 65°C to determine the dry weights of the aboveground and belowground portions. The molybdenum blue technique was used to measure the total P concentration of *L. corniculatus* (Chapman and Pratt, 1962). Fresh leaves and roots were scanned with an Epson Perfection V800 Photo scanner, and the resulting images were analyzed with WinFOLIA Pro 2015 and WinRHIZO Pro 2015 software (Regent Instructions, Canada Inc.) to obtain leaf indicators (surface area, length, width, length/width and perimeter) and root indicators (total length, total surface area, diameter, volume, tip number and hair number), respectively.

### Evaluation of low-P tolerance

An evaluation of the low-P tolerance of different *L. corniculatus* lines was conducted. Low-P tolerance coefficients (based on root morphology, leaf morphology, biomass, plant height and whole plant P concentration of the different *L. corniculatus* lines under low-P stress) were first calculated, followed by a comprehensive evaluation using principal component analysis and affiliation function for low-P tolerance to eliminate differences related to the accessions (Zhu, 2018). The equations were as follows.


(1) Low−P tolerance factor=LP·Xi/NP·Xi

(2) $$\mu (X_{j})=(X_{j}-X_{min})/(X_{max}-X_{min})\;j=1,2,3....,n$$

(3) $$W_{j}=P_{j}/\sum_{j=1}^{m}P_{j}\;j=1,2,3....,n$$

(4) $$D=\sum_{j=1}^{n}\left[\mu (X_{j})\times W_{j}\right]\;j=1,2,3....,n$$

Note that *Xi* denotes the mean value of an indicator, μ(*X_j_*) denotes the affiliation function value of the *j*th composite indicator, *X_j_* denotes the value of the *j*th composite indicator, *X_min_* denotes the minimum value of the *j*th composite indicator, *X_max_* denotes the maximum value of the *j*th composite indicator, *W_j_* denotes the weight of the *j*th composite indicator to all composite indicators, and *P_j_* denotes the contribution of the *j*th composite indicator. D denotes the composite evaluation value of the low-P tolerance of each *L. corniculatus* line.

### Sand culture experiment using the two selected lines

The experimental design of the two screened *L. corniculatus* lines (01549 and 08518) was carried out according to the line Selection part, with 15 seedlings per pot and 3 replicates per treatment. After 7 d of low phosphorus stress, the seedlings were removed from the seedling tray, the roots were washed with distilled water and fresh tissue parts were obtained for the determination of physiological indexes and transcriptome sequencing.

### Determination of physiological indices

Physiological indices, including MDA, soluble protein (SP), proline (Pro) and soluble sugar (SS) contents, as well as SOD, POD, CAT and acid phosphatase (ACP) activities, were determined using an assay kit from Beijing Solarbio Technology Co., Ltd., China.

### Transcriptome sequencing and gene expression profiling

The methods for transcriptome sequencing and gene expression profiling were the same as those described in our previous study (Zhao et al., 2022c). Briefly, total RNA was extracted from the root samples collected from lines 01549 and 08518, and cDNA libraries were constructed, followed by sequencing using the Illumina NovaSeq6000 platform with a PE150 sequencing strategy. Raw reads obtained from sequencing were quality controlled, and low-quality reads were removed. The quality-controlled clean reads were mapped to the *L. corniculatus* reference genome (Ensembl, http://www.kazusa.or.jp/lotus/summary3.0.html) using Bowtie2, and then the RSEM was used to count the number of reads mapped to each reference genome for each sample and to calculate the FPKM (fragments per kilobase of transcript per million mapped reads) for each gene. Paired-end reads from the same fragment were counted as one fragment to obtain the expression levels of genes and transcripts. A differential expression analysis was performed using the R package EdgeR. Genes with FDR (false discovery rate)< 0.05 and |log2FC (fold change)| >1 were defined as differentially expressed genes (DEGs). The GO enrichment analysis is an international standard classification system for gene function. DEGs were functionally annotated using the Gene Ontology database (http://www.geneontology.org/), and GO terms with FDR ≤ 0.05 were selected as significantly enriched GO entries for analysis. Functional annotation of DEGs was performed using the same approach with the KEGG database (http://www.genome.jp/kegg/pathway.html) to analyze pathways significantly correlated with DEGs. The number of genes in each pathway was then counted, and the significantly enriched pathways were analyzed using hypergeometric tests.

### qRT-PCR validation

Eight DEGs (4 upregulated and 4 downregulated) were randomly selected for the experiment using the RNA obtained as described in **Table S3** as the template to confirm the accuracy of the RNA-seq results. cDNA was synthesized from the same RNA samples used for transcriptome sequencing. We performed qRT-PCR on a CFX Connect™ Real-Time System (Applied Biosystems) using UltraSYBR mixture (CWBiotech). The thermocycle parameters were 10 min at 95°C, 40 cycles of 10 s at 95°C, 30 s at 60°C, and 32 s at 72°C, followed by 15 s at 95°C, 1 min at 60°C, 15 s at 95°C, and 15 s at 60°C, in a 20 μl reaction mixture (Zhao et al., 2022c). As an internal reference for normalization, the *Lotus corniculatus*-actin gene was used. Each sample was analyzed in triplicate.

### Statistical analysis

Data were analyzed using Student’s t test in SPSS 25.0 (IBM, Chicago, IL, USA), with a significance level of p<0.05. Relative root length=root length of plants under low-P stress/root length of the control plants, and all morphological and physiological parameters were obtained using similar methods. All data are presented as the means ± standard errors (SE) of three replicates and were plotted graphically using Origin 2021 software.

## Results

### Screening of the low-P-tolerant line (01549) and the low-P-intolerant line (08518)

Previous studies have shown that differences in plant line tolerance to low-P are due to their increased ability to acquire P. (Zhang J. et al., 2019). We speculate that low-P-tolerant plants will have higher P concentration under low-P treatment than low-P-intolerant plants. Thirteen *L. corniculatus* lines were cultured with the LP and NP treatments, which showed the whole-plant P concentration of all 13 *L. corniculatus* lines were significantly deceased (*P*< 0.05) in the range of 9.09%-140.74%, with 08518 (140.74%), 00116 (128.57%), and 01885 (85.71%) showing larger reductions and 01549 showing the smallest reduction (9.09%) under low phosphorus stress (**Table S2**). The value of the affiliation function of each composite indicator was computed using the formula, and the weight of the indicator was computed according to the contribution rate of each indicator. Finally, the comprehensive value of the low-P tolerance of each accession was obtained. The low-P tolerance of different *L. corniculatus* lines varied, where the low-P-tolerance line 01549 was strongest, and the low-P-intolerant line 08518 was weakest (**Table S3**). Therefore, we selected 01549 and 08518 as the low-P-tolerant and low-P-sensitive lines, respectively, for subsequent analyses. At the same time, the root morphology of each accession was examined. The relative P concentration in whole plants was significantly positively correlated with the relative length, relative volume and relative surface area of roots (**Table 1**), and most values of root morphology differed significantly between lines 01549 and 08518 under low phosphorus stress (**Table 2**).

**Table 1:** Correlation analysis of root morphology and low-P tolerance.

	Relative root length	Relative root surface area	Relative root volume	Relative average root diameter	Relative root tip numbers	Relative root dry weight	Relative dry weight of the aboveground portion	Relative P concentration in the whole plant
Relative root length	1							
Relative root surface area	0.948**	1						
Relative root volume	0.907**	0.955*	1					
Relative average root diameter	-0.064	0.149	0.244	1				
Relative root tip numbers	0.114	-0.066	-0.111	-0.554*	1			
Relative root dry weight	0.681*	0.792**	0.786**	0.492	-0.402	1		
Relative dry weight of the aboveground portion	0.084	0.127	0.123	0.559*	-0.386	0.208	1	
Relative P concentration in the whole plant	0.695**	0.634*	0.653*	0.225	0.059	0.518	0.341	1

One and two asterisks indicate significant differences and highly significant differences between the LP and NP treatments, respectively (p< 0.05 and p< 0.01, respectively).

**Table 2:** Effects of *L. corniculatus* lines on root morphology under low phosphorus stress.

P concentration	Accession	Total root length (cm)	Total root surface area (cm^2^)	Root volume (cm^3^)	Root diameter (cm)	Root tips
NP (0.5 mmol)	01549	175.14 ± 7.67b	30.89 ± 2.48b	0.44 ± 0.01b	0.54 ± 0.02a	377.67 ± 8.39c
	08518	171.49 ± 2.12b	28.45 ± 2.61b	0.38 ± 0.02b	0.53 ± 0.04a	423.00 ± 6.00b
LP (0.005 mmol)	01549	217.24 ± 6.56a	34.01 ± 1.20a	0.48 ± 0.02a	0.53 ± 0.03a	541.67 ± 7.77a
	08518	118.34 ± 1.82c	19.85 ± 0.14c	0.25 ± 0.04c	0.48 ± 0.01a	289.67 ± 1.53d

Values are means ± standard errors (n = 3). Different lowercase letters indicate significant differences (P< 0.05) within each index.

### Physiological and biochemical changes

The MDA, Pro, SS, and SP contents and SOD, POD, CAT and ACP activities of both lines 01549 and 08518 increased following LP treatment compared with NP treatment. Compared with those of line 01549, the MDA and SS contents and CAT activity of line 08518 increased under low-P stress, but only the MDA content significantly increased. The Pro and SP contents and SOD, POD and ACP activities of line 08518 were decreased under low-P stress, and the Pro content and SOD, POD and ACP activities were significantly decreased (**Table 3**).

**Table 3:** Effect of low-P stress on the root physiological traits of *L. corniculatus* lines 01549 and 08518.

Accession	P concentration	MDA content (nmol·g^-1^)	Pro content (µg·g^-1^)	SS content (mg·g^-1^)	SP content (mg·g^-1^)	SOD activity (U·g^-1^·min^-1^)	POD activity (U·g^-1^·min^-1^)	CAT activity (U·g^-1^·min^-1^)	ACP activity (μmol·h^-1^·g^-1^)
01549	NP	53.75 ± 0.98d	92.28 ± 0.52c	47.84 ± 0.34b	23.93 ± 0.57c	47.15 ± 0.89c	5480.24 ± 59.89b	840.68 ± 21.06b	61.38 ± 1.11c
	LP	58.01 ± 0.64b	99.74 ± 0.47a	64.33 ± 0.44a	29.04 ± 0.23a	64.84 ± 0.94a	7107.44 ± 57.51a	1465.56 ± 27.24a	100.89 ± 0.83a
08518	NP	55.82 ± 0.44c	88.87 ± 0.69d	50.51 ± 1.77b	26.36 ± 1.21b	36.01 ± 0.41d	2831.97 ± 203.42d	642.81 ± 10.73c	51.46 ± 0.41d
	LP	70.48 ± 0.53a	96.77 ± 0.61b	67.12 ± 2.61a	27.82 ± 0.54a	48.96 ± 0.81b	5158.19 ± 79.51c	1485.19 ± 37.65a	72.57 ± 1.03b

Values are means ± standard errors (n = 3). Different lowercase letters indicate significant differences (P<no><</no> 0.05) within each index.

### DEGs identified in *L. corniculatus*

**Figure 1:**
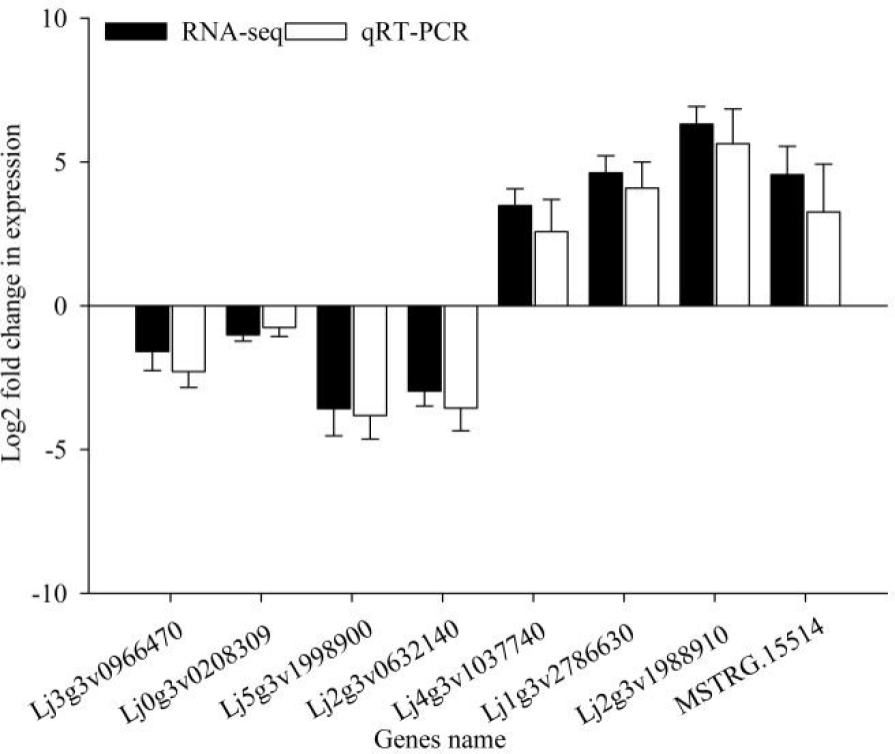
Validation of the transcript abundance of eight selected genes obtained from RNA-seq using qRT-PCR.

Twelve cDNA libraries were sequenced from the roots of lines 01549 and 08518 grown for 7 d under LP and NP treatments using Illumina high-throughput sequencing technology, generating a total of 554 million high-quality clean reads from three replicates of 12 libraries. The sequencing results were submitted to NCBI (**SRA accession: PRJNA871253**), and the Q30 values of the samples reached 93%. Approximately 80% of the total clean reads (Ensembl; http://www.kazusa.or.jp/lotus/summary3.0.html) were mapped to the *L. corniculatus* reference sequence lotus/summary3.0 (**Table S4**). Eight DEGs were randomly chosen for qRT-PCR validation (**Figure 1**). Their expression profiles were significantly associated with the RNA-seq data FPKM, validating the trustworthiness of the transcriptomic data (**Table S5**).

**Figure 2:**
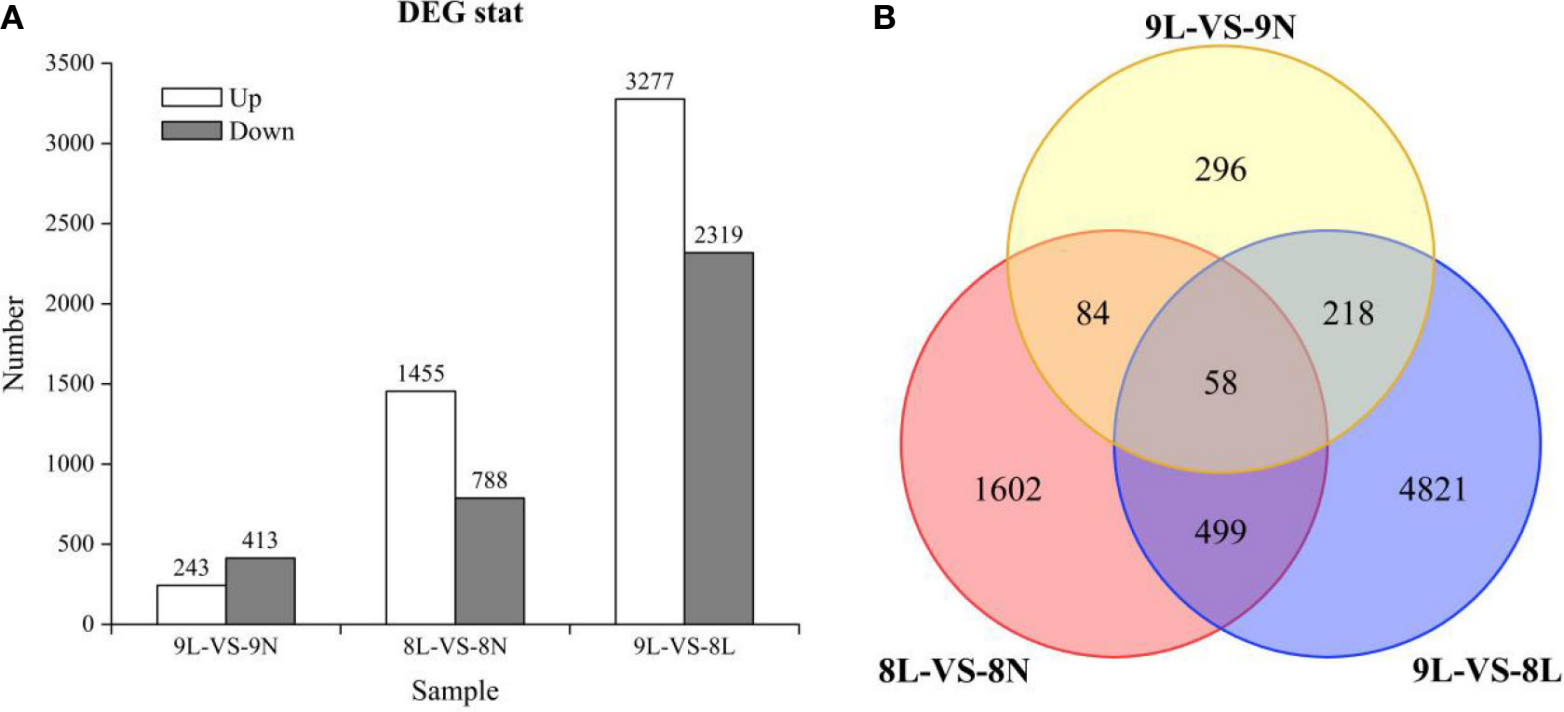
**(A)** The number of diferentially expressed genes in each comparison. **(B)** Venn diagram illustrating the genes of *L. corniculatus* in response to low-P stress.DEGs are shown in Venn diagrams. 9: Low-P-tolerant line 01549; 8: low-P-intolerant line 08518; L: low P; N: normal P.

When the transcripts of *L. corniculatus* were cultivated with the LP treatment 656 DEGs (243 upregulated and 413 downregulated) were identified in line 01549, and 2243 DEGs (1,455 upregulated and 788 downregulated) were identified in line 08518 compared with the NP treatment (**Figure 2**). Surprisingly, the number of DEGs in line 01549 was fewer than that in line 08518, regardless of whether they were upregulated or downregulated. A total of 5,596 DEGs (3,277 upregulated and 2,319 downregulated) were discovered in the low-P-tolerant line 01549 compared with the low-P-intolerant line 08518 under low-P stress. Only 58 common genes were discovered among these DEGs, indicating that these genes were considered valuable candidate genes for improving low-P tolerance (**Figure 2B**).

We performed GO and KEGG pathway enrichment analyses of the DEGs identified in *L. corniculatus*. In total, 278 of the 656 DEGs in line 01549, 1,055 of the 2,243 DEGs in line 08518 and 1,445 of the 5,595 DEGs in the comparison of lines 01549 and 08518 under low-P stress were identified in the GO database. All DEGs identified in the GO database from the three comparison groups were assigned to cellular components, biological processes and molecular functions. In addition, oxidoreductase activity and oxidation-reduction process were the two GO terms with the largest number of DEGs in the three comparison groups. Second, chlorophyllide oxygenase activity, tetrapyrrole binding, enzyme inhibitor activity, endopeptidase inhibitor activity, tetrapyrrole binding and heme binding were also significantly enriched in the three comparison groups (**Table S6**).

**Figure 3:**
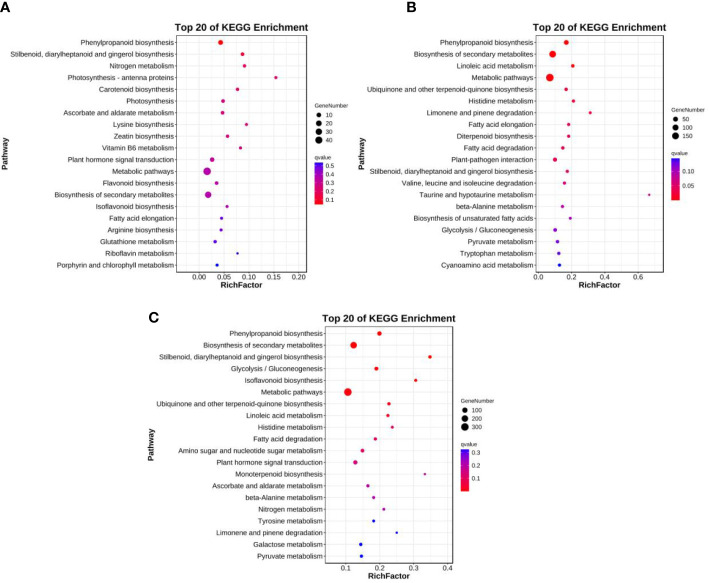
Top 20 enriched KEGG pathways in **(A)** line 01549, **(B)** line 08518, and **(C)** the comparison of lines 01549 and 08518 under low-P stress.

Ten, 24, and 17 significantly enriched pathways were identified among the enriched KEGG pathways for line 01549, 08518, and 01549 vs. 08518 under low-P stress, respectively (**Table S7**). As shown in **Figures 3A-C**, we screened the top 20 enriched KEGG pathways for the analysis of line 01549, line 08518 and lines 01549 vs. 08518 under low-P stress. For line 01549, phenylpropanoid biosynthesis (ko00940, 11 genes), stilbenoid, diarylheptanoid and gingerol biosynthesis (ko00945, 4 genes), and nitrogen metabolism (ko00910, 3 genes) were the three enriched pathways with the highest number of DEGs (**Figure 3A**). For line 08518, phenylpropanoid biosynthesis (ko00940, 43 genes), linoleic acid metabolism (ko00591, 12 genes), and ubiquinone and other terpenoid-quinone biosynthesis (ko00130, 11 genes) were the three main pathways with a large number of DEGs (**Figure 3B**). For the comparison of line 01549 with line 08518, phenylpropanoid biosynthesis (ko00940, 51 genes), stilbenoid, diarylheptanoid and gingerol biosynthesis (ko00945, 16 genes), and glycolysis/gluconeogenesis (ko00010, 30 genes) were the three main pathways (**Figure 3C**). The enriched KEGG pathways therefore revealed that genes in *L. corniculatus* were considerably differentially expressed, suggesting that the various lines activated various molecular mechanisms in response to low-P stress.

### Identification of DEGs in plants under low-P stress

#### DEG-related acid phosphatases and phosphate transporters

**Figure 4:**
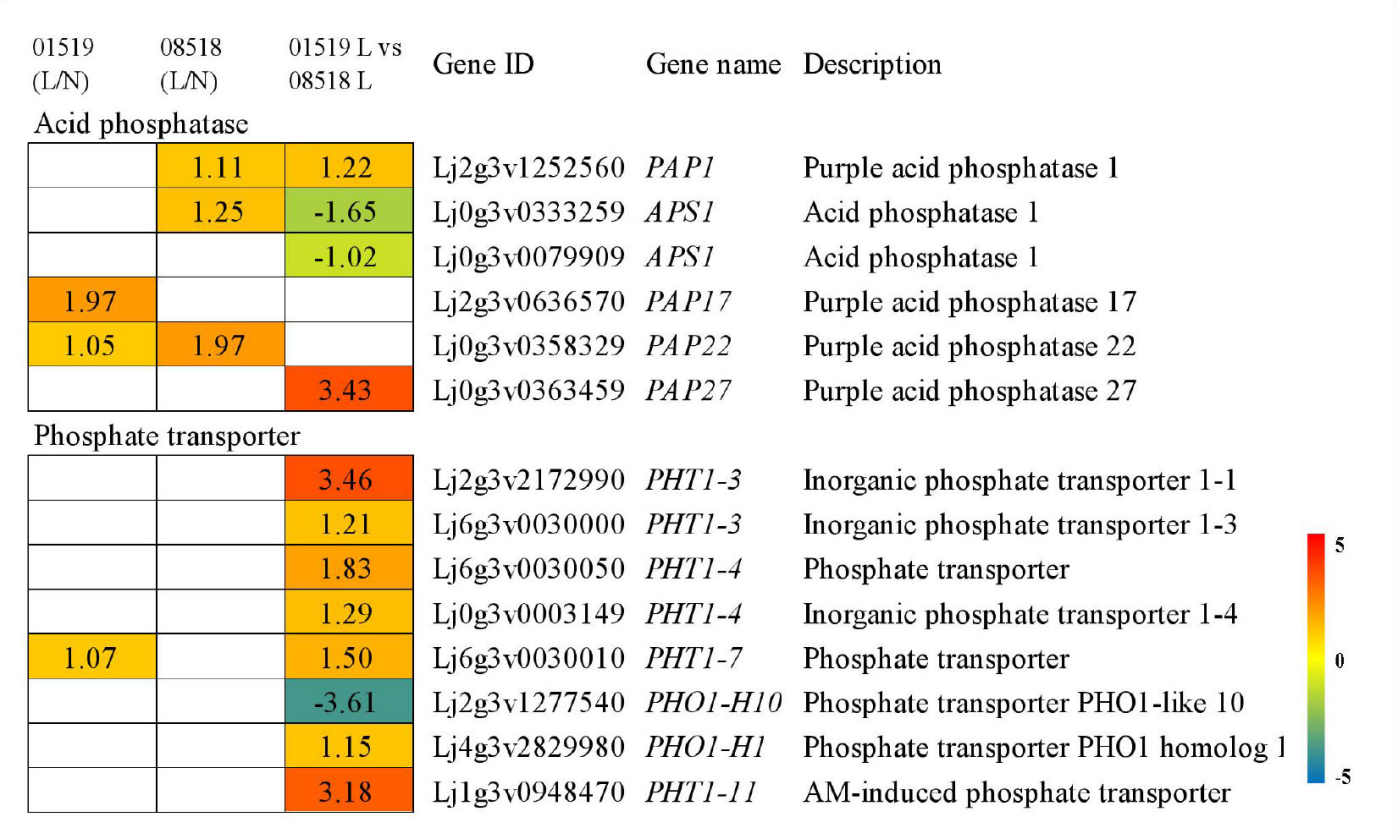
Acid phosphatase and phosphate transporter genes identified in the RNA-seq analysis that showed changes in expression during low-P stress. Log_2_(fold-changes) of the comparisons are shown by the numbers in the gray rectangles. The comparisons show that each of the genes in the picture is significantly differentially expressed.

From the transcriptome data, 6 differentially expressed acid phosphatase genes were identified; among those, 4 *PAP* genes (1 *PAP1*, 1 *PAP17*, 1 *PAP22* and 1 *PAP27*) were significantly upregulated in different lines, but 2 *APS1* genes were downregulated in the comparison of lines 01549 and 08518 under low-P stress. Eight differentially expressed phosphate transporter genes from 6 families were identified; among those, 2 *PHT1-3* genes, 2 *PHT1-4* genes, 1 *PHT1-7* gene, 1 *PHO1-H1* gene, and 1 *PHT1-11* gene were significantly upregulated, but 1 *PHO1-H10* gene was significantly downregulated in line 01549 compared with line 08518 under low-P stress (**Figure 4** and **Table S8**).

#### DEGs related to plant hormone signal transduction

**Figure 5:**
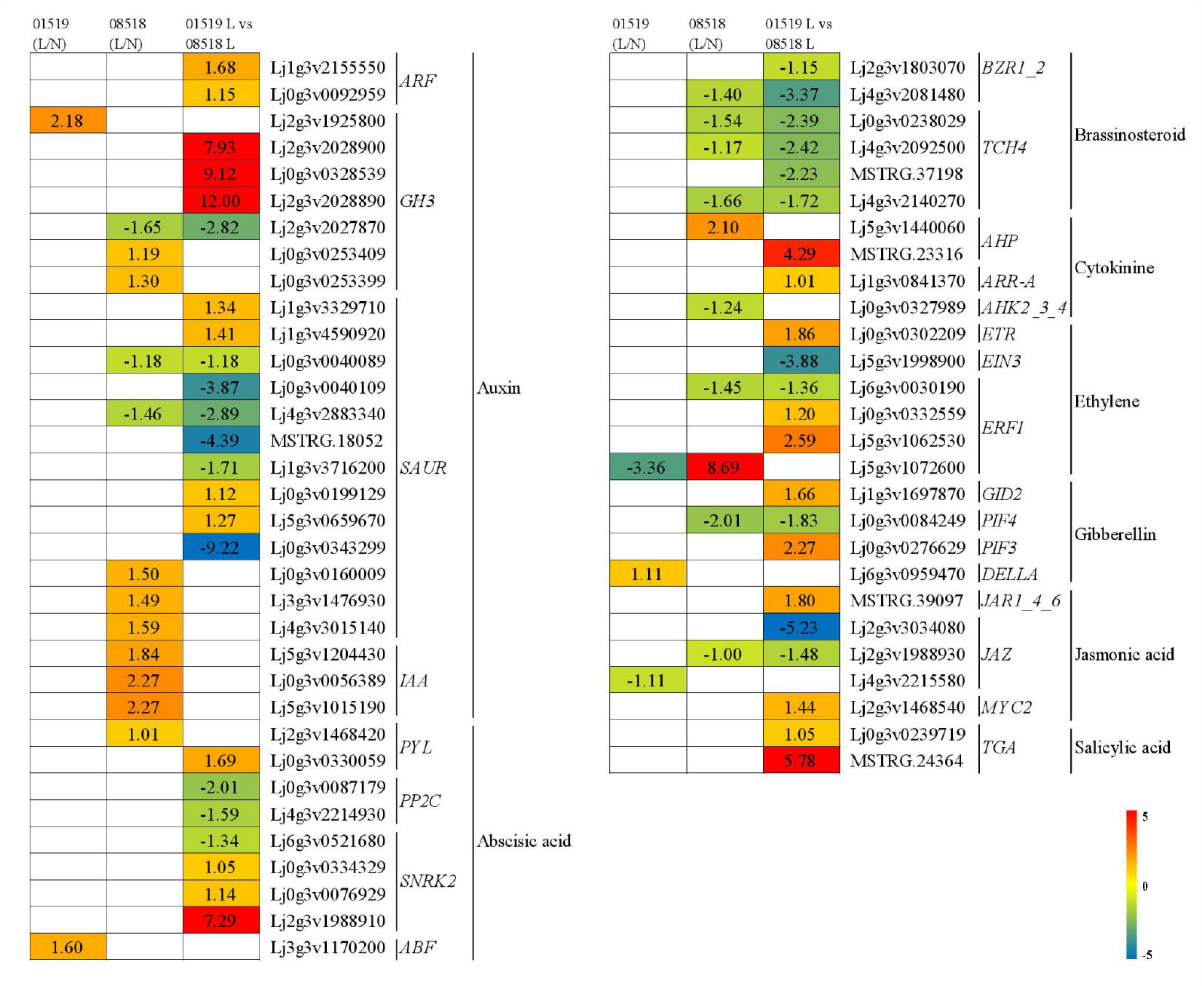
Plant hormone signal transduction-related genes identified in the RNA-seq analysis that showed changes in expression during low-P stress. Log_2_(fold-changes) of the comparisons are shown by the numbers in the gray rectangles. The comparisons show that each of the genes in the picture is significantly differentially expressed.

Sixty-one DEGs, including genes involved in auxin, abscisic acid, brassinosteroid, cytokinin, ethylene, gibberellin, jasmonic acid, and salicylic acid signal transduction, were discovered to be associated with plant hormones (**Figure 5** and **Table S9**). In the auxin signaling pathway, 2 *ARF*, 3 *GH3* and 4 *SAUR* genes were significantly upregulated, but 1 *GH3* and 6 *SAUR* genes were significantly downregulated in line 01549 compared with line 08518 under low-P stress. Two *GH3*, 3 *SAUR* and 3 *IAA* genes were significantly upregulated, but 1 *GH3* and 2 *SAUR* genes were significantly downregulated in line 08518 under low-P stress. One *GH3* gene was significantly upregulated in line 01549 under low-P stress. In the abscisic acid signaling pathway, 1 *PYL* and 3 *SNRK* genes were significantly upregulated, while 2 *PP2C* and 1 *SNRK* genes were significantly downregulated in line 01549 compared with line 08518 under low-P stress. One *ABF* gene and 1 *PYL* gene were significantly upregulated in lines 01549 and 08518, respectively. In the brassinosteroid signaling pathway, 2 *BZR1_2* and 4 *TCH4* genes were significantly downregulated in line 01549 compared with line 08518 under low-P stress, and 1 *BZR1_2* and 3 *TCH4* genes were significantly downregulated in line 08518 under low-P stress. In the cytokinin signaling pathway, 1 *AHP* and 1 *ARR-A* gene were significantly upregulated in line 01549 compared with line 08518 under low-P stress, and 1 *AHP* gene was significantly upregulated and 1 *AHK2_3_4* gene was significantly downregulated in line 08518 under low-P stress. In the ethylene signaling pathway, 1 *ETR* and 2 *ERF1* genes were significantly upregulated, while 1 *EIN3* and 1 *ERF1* gene were significantly downregulated in lines 01549 and 08518 under low-P stress. One *ERF1* gene was significantly upregulated, while 1 *ERF1* gene was significantly downregulated in line 08518 under low-P stress. One *ERF1* gene was significantly downregulated in line 01549 under low-P stress. In the gibberellin signaling pathway, 1 *GID2* and 1 *PIF3* gene were significantly upregulated, while 1 *PIF4* gene was significantly downregulated in lines 01549 and 08518 under low-P stress, 1 *PIF4* gene was significantly downregulated in line 08518 under low-P stress, and 1 *DELLA* gene was significantly upregulated in line 01549 under low-P stress. In the jasmonic acid signaling pathway, 2 *JAZ* genes were significantly downregulated, while 1 *MYC2* and 1 *JAR1_4_6* gene were significantly upregulated in line 01549 compared with line 08518 under low-P stress, 1 *JAZ* gene was significantly downregulated in line 08518 under low-P stress, and 1 *JAZ* gene was significantly downregulated in line 01549 under low-P stress. In the salicylic acid signaling pathway, 2 *TGA* genes were significantly upregulated in line 01549 compared with line 08518 under low-P stress.

#### DEGs related to the biosynthesis of secondary metabolites

**Figure 6:**
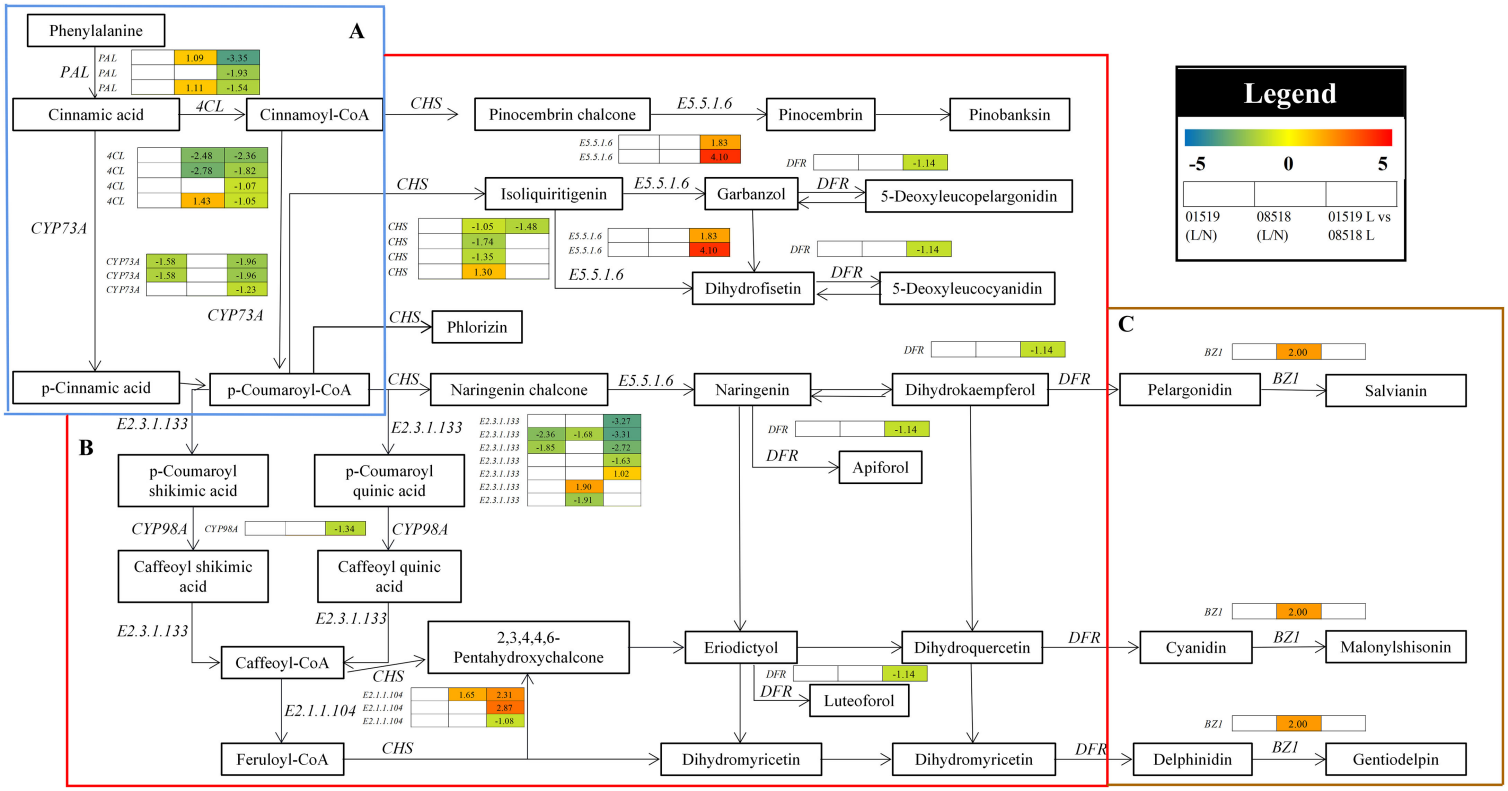
The pathways of phenylpropanoid biosynthesis **(A)**, flavonoid biosynthesis **(B)** and anthocyanin biosynthesis **(C)** enriched in the KEGG analysis. Log2(fold-changes) of the comparisons are shown by the numbers in the gray rectangles. The comparisons show that each of the genes in the picture is significantly differentially expressed.

In the phenylpropanoid, flavonoid and anthocyanin biosynthesis pathways, 29 genes, including *PAL*, *CYP73A*, *4CL*, *CHS*, *E5.5.1.6*, *DFR*, *E2.3.1.133*, *CYP98A*, *E2.1.1.104* and *BZ1*, were differentially accumulated in the *L. corniculatus* roots under low-P stress (**Figure 6** and **Table S10**). Overall, the majority of the structural genes implicated in the pathways were significantly downregulated under low-P stress, repressing secondary metabolism in *L. corniculatus* roots. Among them, 3 *PAL*, 3 *CYP73A*, 4 *4CL*, 1 *CHS*, 1 *DFR*, 4 *E2.3.1.133*, 1 *CYP98A* and 1 *E2.1.1.104* genes were significantly downregulated, while 2 *E5.5.1.6*, 1 *E2.3.1.133* and 2 *E2.1.1.104* genes were significantly upregulated in line 01549 compared with 08518 under low-P stress. 2 *4CL*, 3 *CHS* and 2 *E2.3.1.133* were significantly downregulated, while 2 *PAL*, 1 *4CL*, 1 *CHS*, 1 *E2.3.1.133*, 1 *E2.1.1.104* and 1 *BZ1* genes were significantly upregulated in line 08518 under low-P stress. Two *CYP73A* and *2 E2.3.1.133* genes were significantly downregulated in line 01549 under low-P stress. These results imply that the defense mechanisms of *L. corniculatus* against low-P stress involved the induction of phenylpropanoid and secondary metabolism.

#### DEGs related to carbohydrate metabolism

**Figure 7:**
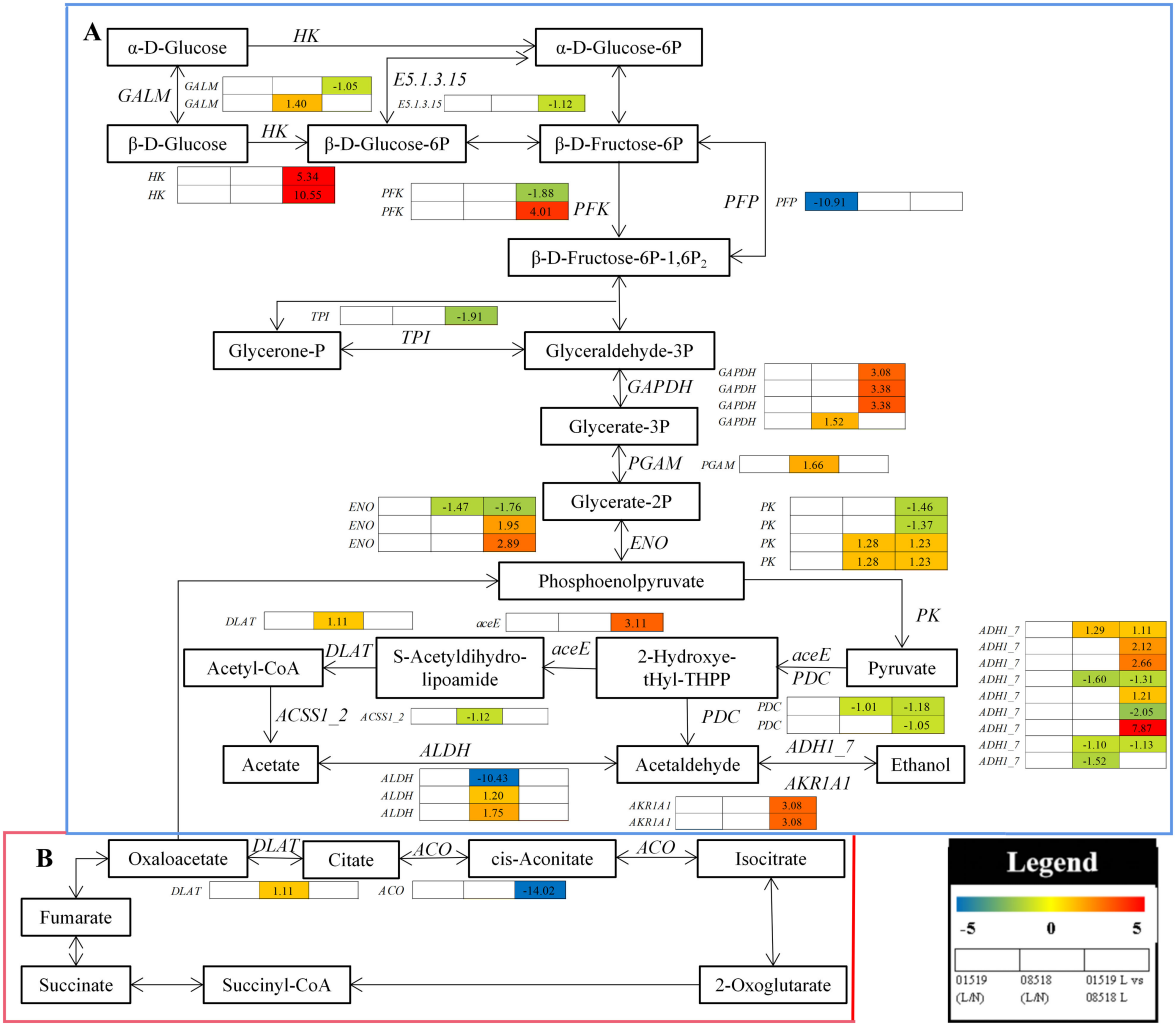
Analysis of the carbohydrate metabolism pathway in plants under low-P stress. **(A)** Glycolysis/gluconeogenesis. **(B)** Citrate cycle. Log_2_(fold-changes) of the comparisons are shown by the numbers in the gray rectangles. The comparisons show that each of the genes in the picture is significantly differentially expressed.

In the glycolysis/gluconeogenesis and citrate cycle pathways, 41 enzyme genes, including *HK*, *GALM*, *E5.1.3.15*, *PFK*, *TPI*, *GAPDH*, *ENO*, *PK*, *aceE*, *PDC*, *ADH1_7*, *PGAM, DLAT, ACSS1_2, ALDH, AKR1A1, PFP* and *ACO*, were differentially accumulated in *L. corniculatus* roots under low-P stress (**Figure 7** and **Table S11**). Among them, 2 *HK*, 1 *PFK*, 3 *GAPDH*, 2 *ENO*, 2 *PK*, 4 *ADH1_7* and 2 *AKR1A1* genes were significantly upregulated, while 1 *GALM*, 1 *E5.1.3.15*, 1 *PFK*, 1 *TPI*, 1 *ENO*, 2 *PK*, 2 *PDC*, 3 *ADH1_7* and 1 *ACO* genes were significantly downregulated in line 01549 compared with line 08518 under low-P stress. 1 *GALM*, 1 *GAPDH*, 2 *PK*, 1 *ADH1_7, 1 PGAM*, 1 *DLAT* and 2 *ALDH* were significantly upregulated, while 1 *ENO*, 1 *PDC*, 3 *ADH1_7*, 1 *ACSS1_2* and 1 *ALDH* genes were significantly downregulated in line 08518 under low-P stress. The 1 *PFP* gene was significantly downregulated in line 01549 under low-P stress. These results imply that the reaction of *L. corniculatus* to low-P stress was mediated by activating the citrate cycle pathway and glycolysis/gluconeogenesis as defense mechanisms.

## Discussion

Plant response to low-P stress is a complex process regulated by multiple genes interacting with each other, and therefore, manipulation of a set of genes or individual genes is not sufficient to study plant tolerance to low-P (Sun et al., 2021; Zhao et al., 2022b). In this study, two selected genotypes (01549 and 08518) of *L. corniculatus* seedlings to better highlight the differences in resistance between the two *L. corniculatus* genotypes by systematically studying the morphological, physiological, and transcriptional responses of *L. corniculatus* to low-P stress. In this study, low phosphorus stress caused extensive changes in morphological traits, physiological indicators, and gene expression in the *L. corniculatus* roots.

In the study, low-P stress increased the MDA content in the roots of both lines 01549 and 08518 compared to the NP treatment, and the MDA content of line 01549 was significantly lower than that of line 08518 during low-P treatment. Based on these results, low-P stress caused a large number of superoxide radicals to be produced in the *L. corniculatus* roots, which caused an imbalance in plant metabolic oxygen production and subsequent oxidative damage to the plant (Tarumoto et al., 2022). However, the membrane system of line 08518 was damaged to a greater extent by low-P stress. Plants scavenge the accumulated O^2-^ through the antioxidant enzyme system consisting of SOD, CAT and POD to avoid cell membrane damage and keep the free radicals in plants at a low level under adverse conditions (Niu et al., 2013; Xu et al., 2018). In this study, we found that the antioxidant enzyme activities in both lines 01549 and 08518 were increased to enhance the adaptation to the low-P environment. Osmoregulatory substances in plants play an important role in reducing plant damage caused by stress(Anawar et al., 2018). In this study, we found that the proline, soluble sugar and soluble protein contents in lines 01549 and 08518 were increased under low phosphorus stress, indicating that the root cells of *L. corniculatus* were able to reduce the intracellular osmotic potential by regulating the content of their organic osmoregulatory substances, thus achieving self-protection and enhancing the tolerance to low phosphorus (Sun and Zheng, 2022). However, the antioxidant enzyme activities and osmoregulatory substance contents of line 01549 was higher than that of line 08518, indicating that line 01549 was more adapted to the low-P environment. Meanwhile, these data show very small differences in some cases between the two treatments in P treatment concentrations, this could be the different phosphorus uptake capacity and phosphorus utilization efficiency of different lines of *L. corniculatus*. However, the exact reasons need further analysis.

By studying the transcriptional responses of *L. corniculatus* to low-P stress, we have revealed different patterns of *L. corniculatus* to response low-P stress, including carbohydrate metabolism, acid phosphatases and phosphate transporters, biosynthesis of secondary metabolites, and plant hormone signal transduction.

Anthocyanin biosynthesis is catalyzed by phenylpropanoid and flavonoid biosynthetic pathways through related genes such as *PAL*, *CHS*, *CHI*, *F3H*, *DFR*, and *ANS* (Liu et al., 2016; Farooq et al., 2016). The expression of these genes involved in secondary metabolite biosynthesis were differentially expressed in *L. corniculatus* lines under low-P stress in the present study, with *PAL*, *4CL*, *CHS* and *DFR* expression downregulated in line 01549 compared with line 08518 under low-P stress, while line 08518 showed upregulated *PAL* expression and decreased total gene expression of the *4CL* and *CHS* genes in the anthocyanin biosynthetic pathway under low-P stress, and the *BZ1* gene was upregulated in line 08518 under low-P stress. Although the expression of these genes does not fully reflect the antioxidant characteristics of *L. corniculatus* and requires further verification in cells and *in vivo* tissues, it nevertheless provides an indication of the tolerance of *L. corniculatus*. Regardless of the comparative group, line 08518 was more sensitive to low-P stress, suggesting that a low P concentration may have suppressed gene transcription or translation in *L. corniculatus*.

Acid phosphatase and high-affinity phosphate transporter family genes play key roles in the activation of phosphorus uptake and efficient phosphorus transport under low-P stress (Kuerban et al., 2020). Phosphatase is an adaptation-inducible enzyme that promotes the mineralization and decomposition of soil organic P (Zhu et al., 2020). In our study, two acid phosphatase 1 (*APS1*) genes were downregulated in lines 01549 and 08518 under low-P stress, and four purple acid phosphatase (*PAP*) genes were significantly upregulated by low-P stress. Fita et al. (2012) found that *Cm-PAP10.1* and *Cm-PAP10.2*, genes encoding purple acid phosphatases, were upregulated in melon under low-P stress, indicating that the activity of APS secreted by plant roots under low-P stress was positively correlated with the degree of P deficiency. Meanwhile, genes encoding phosphorus transporter proteins were screened in transcriptome data, among which eight DEGs of high-affinity phosphorus transporter systems (*Pht1* and *Pho1* families) were screened, all of which were differentially expressed to varying degrees in the two *L. corniculatus* lines treated with low P. Hu et al. (2021) found that the *PHO1* and *PHT1* genes were upregulated in the roots of *Zygophyllum xanthoxylum*, suggesting that these phosphorus transporter protein genes may play a vital role in regulating the distribution, transport and maintenance of dynamic homeostasis of P in the plant body during P deficiency (Hu et al., 2021), but their functions in response to low-P stress remain to be further investigated.

Several respiratory metabolic pathways have been identified in plants, including the glycolytic pathway (EMP), the tricarboxylic acid cycle (TCA), and the mitochondrial electron transport chain (Van Dongen et al., 2011). Plants produce ATP and CO_2_ through the breakdown of photosynthetic products (e.g., glucose) by respiration to promote root growth and development or maintain root activity for nutrient and water uptake and translocation (Chen et al., 2021). The differential expression of 41 key glycolysis and TCA cycle-related genes was discovered in this study. Among these genes, the glycolytic pathway-related genes *HK, PFK, GAPDH, ENO, PK, ADH1 7*, and *AKR1A1* were elevated, whereas the TCA cycle-related aconitate hydratase gene *ACO3* was downregulated in line 01549 compared with line 08518 under low-P conditions. The expression of some genes related to the glycolytic pathway, namely, *GALM*, *PK*, *ADH1_7*, and *ALDH*, was downregulated, and the expression of *DLAT*, a pyruvate dehydrogenase E2 component of the TCA cycle, was upregulated in line 01549 compared with line 08518 under low-P stress, suggesting that low-P stress suppresses both pathways while increasing electron transport, and the *L. corniculatus* root maintains an ATP supply by constantly changing the transcript levels of genes encoding critical enzymes in the glycolytic/gluconeogenic pathway. (Du et al., 2020).

Low-P stress affects the synthesis and distribution of hormones in plants, which further regulates root conformation, such as root morphology and structure (Zhang Z. et al., 2014; Kumar et al., 2021; Martínez-Andújar et al., 2017). In this study, the expression genes involved in plant hormone signal transduction was significantly changed under low-P conditions, which may indicate that hormones play a crucial role in root development under low-P conditions (Zhang X. et al., 2019; Bhosale et al., 2018). In our study, the expression of the *ARF*, *GH3*, *PYL*, *SNRK2*, *ABF*, *AHP*, *ARR-A*, *ERF1*, *GID*, *PIF3*, *JAR1_4_6*, *MYC2* and *TGA* genes involved in in auxin, abscisic acid, cytokinin, ethylene, gibberellin, jasmonic acid and salicylic acid signaling, respectively, was upregulated in line 01549 compared with line 08518 under low-P stress, suggesting that these genes associated with phytohormone signaling are involved in regulating the root morphology of *L. corniculatus* in response to low-P stress. Gene expression was increased in line 01549, and the values of root morphological indicators were significantly higher in line 01549 than in line 08518, indicating that line 01549 regulates root development more quickly in response to stress. Brassinosteroids (BRs) are plant hormones that promote cell elongation and division and play an important role in plant growth and development (Planas-Riverola et al., 2019). In our study, the expression of all genes related to brassinosteroid signaling was downregulated, potentially due to the negative regulation of brassinosteroids in response to low-P stress in the root, but further analyses are needed to determine the exact cause. Similarly, transcription factors related to hormone metabolism and signal transduction play a crucial role. Studies have shown that the growth hormone-responsive transcription factor SAUR also regulates root morphology and promotes lateral root development (Xie et al., 2000). *Arabidopsis* RLK (receptor-like protein kinase) regulates root hair development (Wei and Li, 2018). In this study, *SAUR* genes were both upregulated and downregulated in line 01549 compared with line 08518 under low-P stress, indicating that both lines suffered from low-P stress, regardless of the accession. Two *SAUR* genes were downregulated in line 08518, and no difference in *SAUR* expression was identified in line 01549 under low-P stress, indicating that line 08518 has a reduced root morphology and requires the regulation of *SAUR* genes to respond to low-P stress.

By determining morphological and physiological features, we verified that the phosphorus-resistant genotype 01549 performed better under low-P stress, which was also highly supported by transcriptome data. However, previous studies have shown that these genes do not always coincide with changes in physiological indicators, and it is possible that other unknown genes are involved in the response of *L. corniculatus* to low-P stress.

## Conclusions

Key KEGG pathways and their candidate genes were evaluated to explore changes in the morphological and physiological characteristics of the two *L. corniculatus* lines under low-P stress. We found that the root morphology of phosphorus-tolerant line 01549 increased and that of phosphorus-sensitive line 08518 decreased under low-P stress. *L. corniculatus* adapted to the low-P environment by increasing enzyme activity and the contents of osmoregulatory substances to rapidly regulate its physiological and metabolic functions as a mechanism to mitigate the injury. In addition, a multilevel analysis of changes in gene expression in *L. corniculatus* was conducted. Low-P stress activated important pathways, such as plant hormone signal transduction, and we identified key candidate genes, such as *PAP*, *PHT1* and *PHO1*. The combination of morphological, physiological, and transcriptome data reported in this work provides a theoretical foundation for future studies of the complex processes driving low-P responses in *L. corniculatus* and other plant species.

## Data availability statement

The data presented in the study are deposited in the BCBI repository, accession number PRJNA870253.

## Author contributions

XZ and K-KC: experimental design, experimental performance, experimental data collection, data analysis, manuscript writing and revision. L-LZ: seed provision, manuscript writing, resource provision and funding acquisition. L-TW: experimental performance, experimental data collection and data analysis. P-CW: manuscript writing and revision. All authors contributed to the article and approved the submitted version.
